# Home blood sodium monitoring, sliding-scale fluid prescription and subcutaneous DDAVP for infantile diabetes insipidus with impaired thirst mechanism

**DOI:** 10.1186/1687-9856-2012-18

**Published:** 2012-06-09

**Authors:** Shihab Hameed, Abel C Mendoza-Cruz, Kristen A Neville, Helen J Woodhead, Jan L Walker, Charles F Verge

**Affiliations:** 1 Endocrinology, Sydney Children’s Hospital, Randwick, Australia; 2 School of Women’s and Children’s Health, UNSW, Sydney, Australia; 3 Paediatric Endocrinologist, Sydney Children’s Hospital, Senior Research Fellow (Lecturer), UNSW, High Street, Randwick, NSW 2031, Australia

**Keywords:** Diabetes insipidus, Thirst, Fluid Therapy, Water-electrolyte imbalance, Hypothalamic diseases

## Abstract

**Background/Aims:**

Infants with diabetes insipidus (DI), especially those with impaired thirst mechanism or hypothalamic hyperphagia, are prone to severe sodium fluctuations, often requiring hospitalization. We aimed to avoid dangerous fluctuations in serum sodium and improve parental independence.

**Methods:**

A 16-month old girl with central DI, absent thirst mechanism and hyperphagia following surgery for hypothalamic astrocytoma had erratic absorption of oral DDAVP during chemotherapy cycles. She required prolonged hospitalizations for hypernatremia and hyponatremic seizure. Intensive monitoring of fluid balance, weight and clinical assessment of hydration were not helpful in predicting serum sodium. Discharge home was deemed unsafe. Oral DDAVP was switched to subcutaneous (twice-daily injections, starting with 0.01mcg/dose, increasing to 0.024mcg/dose). The parents adjusted daily fluid allocation by sliding-scale, according to the blood sodium level (measured by handheld i-STAT analyser, Abbott). We adjusted the DDAVP dose if fluid allocation differed from maintenance requirements for 3 consecutive days.

**Results:**

After 2.5 months, sodium was better controlled, with 84% of levels within reference range (135-145 mmol/L) *vs.* only 51% on the old regimen (p = 0.0001). The sodium ranged from 132-154 mmol/L, compared to 120–156 on the old regimen. She was discharged home.

**Conclusion:**

This practical regimen improved sodium control, parental independence, and allowed discharge home.

## Introduction

Infants treated with DDAVP for diabetes insipidus (DI) are prone to wide sodium fluctuations, due to uncertainties in the clinical assessment of hydration status and a high fluid diet [1]. Infants may drink due to hunger rather than thirst, leading to dilutional hyponatremia and seizures. Management is further complicated in those with impaired thirst mechanism and/or hypothalamic hyperphagia. Recently the use of subcutaneous DDAVP [2] and the use of home sodium monitoring [3] have been reported . We aimed to improve sodium control and parental independence using these in combination with a written sliding-scale fluid prescription plan.

## Methods

### Patient history

A 16-month old girl developed central DI, absent thirst mechanism and hypothalamic hyperphagia following surgery for a large hypothalamic pilomixoid astrocytoma (6.6 × 7.1 × 6.7 cm). Postoperatively, she developed profound polyuria (up to 20 ml/kg/hr) and received intravenous vasopressin infusion in the intensive care ward, before switching to oral DDAVP. The oral DDAVP was usually given twice-daily, with the parents awaiting breakthrough diuresis before administration of each dose on an empty stomach. The antidiuretic effect of each dose varied considerably, necessitating frequent dosage changes and supplemental doses at various times of the day and night. The changes in antidiuretic action of DDAVP were most pronounced during chemotherapy cycles, possibly reflecting variable absorption of oral DDAVP from the gastrointestinal tract. Additionally, the need for intravenous fluid hydration for platinum based alkylating chemotherapy further complicated fluid management. Despite intensive monitoring of fluid balance, weight and clinical assessment of hydration by experienced clinicians, the serum sodium fluctuated widely. She was discharged home following prolonged hospitalization for hypernatremia only to require intensive care admission the next day following a hyponatremic seizure. Further discharge home was, therefore, deemed unsafe.

The patient also had anterior hypopituitarism, treated with replacement thyroxine (89mcg/m^2^/day, 4.3mcg/kg/day) and hydrocortisone (10 mg/m^2^/day). She had hypothalamic hyperphagia and caloric intake was limited by the use of a diluted formula. Despite these measures, the weight standard deviation score increased markedly from −2.1 SDS pre-operatively to +2.5 SDS 8 months postoperatively. Sodium intake was maintained at standard requirements.

### New management regimen

#### Sodium monitoring

The hospital laboratory performed sodium measurements using indirect potentiometry (Beckman Coulter, Fullerton, USA). Home sodium monitoring was performed daily by the parents, using a handheld i-STAT analyser (Abbott, USA). Sodium results by i-STAT correspond well with laboratory sodium levels by error grid analysis [3]. The parents collected 95μL of capillary blood onto single use cartridges, and results were available within 6 minutes. We compared sodium results for 3.9 months before and 2.5 months following introduction of the new regimen.

#### DDAVP

DDAVP was switched from oral to subcutaneous (4mcg/ml diluted 1:10 in normal saline, prepared each week by hospital pharmacy in glass vials). We found that this preparation remained stable for at least one week (based on the duration of antidiuretic response). The injections were administered by the parents twice-daily by insulin syringe, starting with 0.01mcg/dose (based on 200-times potency vs. oral), increasing to 0.024mcg/dose (0.003mcg/kg/dose).

#### Fluid prescription by sliding-scale

Daily oral fluid allocation was adjusted by the parents using a sliding-scale according to the blood sodium level. We designed the sliding scale using the formula for water deficit in ml [4], and the parents were supplied with a written plan (Table 1). We adjusted the DDAVP dose if the fluid allocation differed from maintenance requirements for 3 consecutive days. We ceased monitoring urine output, which required labour-intensive weighing of nappies.

**Table 1:** Sliding-scale fluid prescription given to the parents to adjust daily fluid prescription according to the home blood sodium result

**Blood Sodium (mmol/L)**	Daily Fluid Prescription (mls)*	
Below 130	100	Telephone and go to ED for assessment
130-134	500	Repeat sodium level before PM dose
135-139	800	Repeat sodium level before PM dose
140-145	1,200	Sodium is in target range
146-150	1,350	Repeat sodium level before PM dose
151-155	1,500	Repeat sodium level before PM dose
Over 155	1,800	Telephone and go to ED for assessment

Parents were instructed to telephone the Paediatric Endocrine department and attend the emergency department (ED) if levels were below 130 or over 150 mmol/L.

*The fluid prescription is based on 1,200 ml maintenance fluids, with additions or subtractions based on the formula for calculating water deficit in ml (600 × wt(kg) × (1-target [Na]/measured [Na]). If the sodium is >150 mmol/L, the extra fluid above maintenance has been reduced by 25% to avoid excessively rapid correction.

## Results

**Figure 1:**
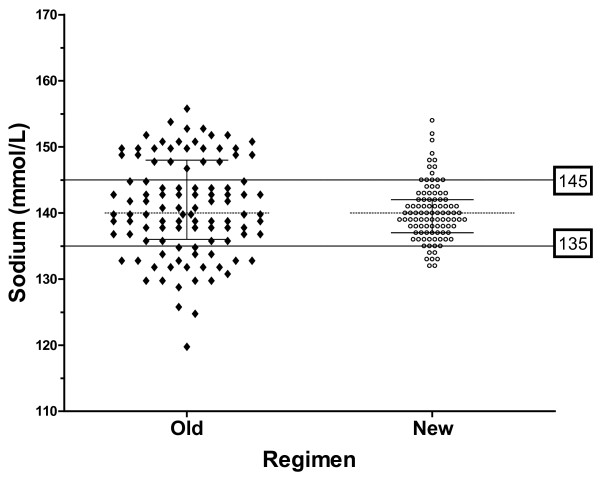
**Scatter plots of sodium levels during 3.9 months on oral DDAVP (n = 106) compared with the subsequent 2.5 months on the new regimen of twice-daily subcutaneous DDAVP with sliding scale fluid prescription, adjusted according to the results of home sodium monitoring (n = 98).** Median and interquartile range for sodium levels on each regimen are also shown.

After 2.5 months, the sodium was better controlled, with 82/98 (84%) within reference range (135-145 mmol/L) *vs*. only 54/106 (51%) on the old regimen (Chi squared test, p = 0.0001). The sodium ranged from 132-154 mmol/L, compared to 120–156 on the old regimen (Figure 1). The standard deviation of sodium levels was lower on the new regimen (4.2 mmol/L *vs.* 7.3 mmol/L on the old regimen).

The patient was successfully discharged home. There were no further hospital admissions due to sodium fluctuations. The parents reported a strong preference for the new regimen. In particular, they were reassured by the ability to determine the sodium level quickly by i-STAT. They also preferred the fixed, twice-daily regimen of DDAVP administration (which avoided the need to administer DDAVP during their sleeping hours), despite the need for subcutaneous injection. Furthermore, they appreciated not having to weigh nappies, which they found to be laborious, unpleasant and difficult to perform when away from home. The family also enjoyed not having to delay feeding following administration of DDAVP, which was distressing to the infant and had been a requirement of oral dosing (in an attempt to improve the reliability of absorption). The i-STAT costs ($10, 000 initially, then $5 per day for consumables) were less than the cost of her previous hospital admissions, including intensive care, for hypo and hypernatremia.

## Discussion

Oral DDAVP absorption may be impaired when given with meals [1,5]. Intranasal DDAVP can be difficult to administer to infants and absorption may be impaired during coryzal illnesses [5]. Rivkees reported that subcutaneous DDAVP is useful when precise and consistent dosing is required [5]. Blanco and colleagues [2] reported a retrospective comparison of 6 infants treated with subcutaneous DDAVP and 4 treated with intranasal lysine vasopressin. They found that subcutaneous dosing resulted in a narrower range of sodium levels and a higher percentage within the normal range. None of their patients had impairment of thirst mechanism and all fed *ad libitum.*

Green and Landt [3] reported a cross-over study involving 4 children with DI and impaired thirst mechnanism (or inability to access water). Home sodium monitoring by i-STAT was compared with routine care (regular laboratory sodium measures weekly to monthly, and additional measures when clinically indicated). All carers preferred iSTAT to routine care, but there was no significant difference in the number of hospitalisations in the two arms. They recorded significantly higher number of telephone calls to the study physician during home sodium monitoring. In contrast, we found that by providing the parents with a sliding scale fluid prescription, they were able to manage the DI without needing to call regularly for advice.

## Conclusion

We describe a practical regimen combining subcutaneous DDAVP and sliding-scale fluid prescription according to home sodium monitoring levels that improved sodium control, increased parental independence, and allowed discharge home.

## Consent

Written informed consent was obtained from the parents of the patient for publication of this Case report and any accompanying images. A copy of the written consent is available for review by the Editor-in-Chief of this journal.

## Competing interests

The authors declare that they have no competing interests.

## Authors’ contributions

SH and CFV contributed to the conception and design of the case report. SH, CMC, KAN, HJW, JLW and CFV all contributed to the acquisition and interpretation of data and to the drafting and revision of the manuscript. All authors read and approved the final manuscript.

## Disclosure summary

Nothing to disclose.
